# Pre-emptive Nebulized Ketamine Versus Lidocaine for Post-tonsillectomy Pain Management in Children: A Comparative Study

**DOI:** 10.7759/cureus.70129

**Published:** 2024-09-24

**Authors:** Manikandan Seetharamaraju, Sridevi Mulimani, Anusha Suntan, Vijaya Sorganvi

**Affiliations:** 1 Anesthesiology, Shri B M Patil Medical College Hospital, Bijapur Lingayat District Educational Association (BLDE) (Deemed to be University), Vijayapura, IND; 2 Community Medicine, Bijapur Lingayat District Educational Association (BLDE) (Deemed to be University), Vijayapura, India

**Keywords:** ketamine, nebulization, pain management, rescue analgesia, tonsillectomy

## Abstract

Background

Tonsillectomy is associated with significant pain, and postoperative pain control is often unsatisfactory. There have been several methods adopted to treat postoperative pain, but none of the methods were effective, with patients continuing to undergo severe postoperative pain. Hence, our study aimed to compare the efficacy of pre-emptive nebulized ketamine versus pre-emptive nebulized lidocaine with a control group receiving nebulized saline for pain control in children undergoing tonsillectomy.

Methods

In this prospective randomized clinical trial, 105 patients with American Society of Anesthesiologists (ASA) Ⅰ and Ⅱ undergoing tonsillectomy were enrolled and randomized into three groups, group K, group L, and group C, with 35 patients in each group, wherein pre-emptive nebulized ketamine, lidocaine, and saline were given to each group of patients, respectively. Faces Pain Scale-Revised (FPS-R), sedation scale scores, and the usage of rescue analgesia were noted postoperatively for the first six hours. Hemodynamic parameters were noted before and after nebulization. The primary objective was to determine the number of patients requiring rescue analgesia as an indicator of postoperative pain control.

Results

In this randomized clinical trial, pre-emptive nebulized ketamine significantly reduced the need for rescue analgesia compared to lidocaine and saline (p<0.05). Only 14.3% (n=5) of patients in the ketamine group required rescue analgesia compared to 85.7% (n=30) in the lidocaine group and 91.4% (n=32) in the control group. Nebulized ketamine given pre-emptively is an effective strategy for reducing postoperative pain in pediatric tonsillectomy.

Conclusion

Pre-emptive nebulized ketamine was found to be effective when compared with lidocaine nebulization in reducing postoperative pain in children undergoing tonsillectomy.

## Introduction

One of the most common surgeries in children is tonsillectomy, and pain management in tonsillectomy remains difficult. Following a tonsillectomy, problems such as acute pain, hypovolemia from reduced oral intake, respiratory depression, and postoperative hemorrhage are common consequences. There are several reasons why pain following a tonsillectomy is not adequately treated, such as the pediatric population being unable to explain their degree of pain properly, the inaccurate dosage of analgesics, and the surgery being thought as minimally intrusive and thus people tend to underestimate the severity of pain [1,2].

Previously, many methods such as infiltration of perioperative local anesthetics, glossopharyngeal blocks, lesser palatine nerve blocks, and the usage of honey have been tried in postoperative pain management in tonsillectomy. However, all those methods were not effective in reducing early postoperative tonsillectomy pain [3,4]. The most widely prescribed analgesics in children are nonsteroidal anti-inflammatory drugs (NSAIDs); nevertheless, because they decrease platelet aggregation and lengthen the bleeding period, they may increase perioperative bleeding. Fear, worry, and a lack of social support are some of the factors that exacerbate children's physical discomfort. Proper evaluation of postoperative pain in pediatric patients can greatly enhance their comfort and overall quality of life [5,6].

Nebulization is mostly used to deliver drugs to patients safely and conveniently, with the added benefit of allowing the medicine to enter their lower respiratory tract. Compressed air breaks the liquid into droplets during nebulization, producing big particles (10-25 micrometers) that mostly settle in the mouth and throat, with smaller particles (5-10 micrometers) settling in the area where the mouth and airway meet [7,8].

Ketamine is a noncompetitive N-methyl-D-aspartate (NMDA) receptor complex antagonist that has been used for decreasing postoperative pain when administered intravenously and to treat sore throats when administered by nebulization because of its anti-nociceptive and anti-inflammatory action. One of the most widely used local anesthetics is lidocaine. Preoperative nebulization reduces the overall amount of opioid analgesia administered after surgery, as well as tube and nasal pack tolerance and airway circulatory reflexes during induction and emergence. It was shown that lidocaine nebulization inhibits the production of neuropeptide by suppressing the excitatory sensory C fibers in the airways [9,10].

While various methods have been explored for managing post-tonsillectomy pain, the use of pre-emptive nebulized ketamine versus lidocaine remains underexplored. This study aims to fill this gap by comparing the efficacy of these two treatments in the pediatric population, providing novel insights into optimal pain management strategies for tonsillectomy. Hence, we have made an analogy between the effectiveness of nebulized ketamine and lidocaine in providing analgesia by analyzing the Faces Pain Scale scores after tonsillectomy and the need for rescue analgesia, which indicates good postoperative pain management.

We hypothesized that nebulized ketamine is more efficacious than nebulized lidocaine in reducing post-tonsillectomy pain in children.

## Materials and methods

This randomized comparative study was started after obtaining clearance from the Ethics Committee of Shri B M Patil Medical College Hospital and Research Centre (reference number: BLDE(DU)/IEC/782/2022-23). This study was conducted for a period of one year from May 2023 to May 2024 in the Department of Anesthesiology, Shri B M Patil Medical College Hospital and Research Centre, BLDE (Deemed to be University), Vijayapura, Karnataka, India. A total of 130 patients underwent tonsillectomy during the study period, and among them, 105 patients were selected. The inclusion criteria were American Society of Anesthesiologists (ASA) Ⅰ and Ⅱ, and age between five and 12 undergoing tonsillectomy. The exclusion criteria were patients having congenital abnormality and patients having a history of any drug allergy. This study had been registered in the Clinical Trials Registry - India (CTRI) with registration number CTRI/2023/04/052113.

Based on a previous study [10], the mean±standard deviation (SD) of time to extubate (minutes) were 7.6±1.3, 6.3±1.43, and 6.1±1.50 in the control group, K-N1 group, and K-N2 group, respectively. Using a type 2 error of 0.05 and a power of 90%, the calculated sample size was 35 per group (i.e., a total sample size of 105 assuming equal group sizes) with an effect size of 0.355 for detecting a difference in means between the three groups. G* power software version 3.1.9.7 was used to calculate the sample size.

The primary objectives of this study were to compare the effects of nebulized ketamine versus lidocaine for postoperative analgesia effect and evaluate the usage and frequency of rescue analgesia according to Faces Pain Scale scores. The secondary objectives were assessment of SpO2, respiratory rate, heart rate, and blood pressure pre- and post-nebulization and observation of side effects such as vomiting, desaturation, and hallucinations.

The patients enrolled in this study were randomly divided into three groups by computer-generated randomization table depending on the pre-emptive nebulized drug. The groups were group K, group L, and group C with each group receiving nebulized ketamine (2 mg/kg), lidocaine (4 mg/kg), and saline (4 mL), respectively, with doses selected based on previous studies demonstrating their efficacy and safety in the pediatric population. A pre-anesthetic checkup was done in the ward, and the Faces Pain Scale was explained to the patient and their attender. Patients were kept nil per oral six hours before surgery. The procedure was explained to the patient attender, and informed consent was obtained. Nebulization has been given by a standard hospital portable nebulizer 15 minutes before the induction of general anesthesia. All the patients' vital parameters, such as SpO2, respiratory rate, heart rate, and blood pressure, were recorded before and after nebulization, which is before the induction of general anesthesia.

The method of anesthesia was the same for all groups. Anesthesia was induced with propofol 2-3 mg/kg, fentanyl 1 microgram/kg, and atracurium 0.5 mg/kg. The size of the endotracheal tube was selected according to age. Anesthesia and muscle relaxants have been maintained with sevoflurane in a 50% oxygen/air mixture and 0.15 mg/kg atracurium at fixed intervals. At the end of the procedure, neuromuscular blockade was reversed with standard doses of neostigmine 0.05 mg/kg and glycopyrrolate 0.008 mg/kg.

After extubating, patients were transported to the post-anesthesia care unit where Faces Pain Scale-Revised (FPS-R) scoring was recorded at 30 minutes and first, second, third, fourth, fifth, and sixth hours postoperatively. Patients received IV paracetamol 15 mg/kg as rescue analgesia if requested, and the total consumption of postoperative rescue analgesia was recorded.

Any postoperative adverse events such as desaturation (<95%), postoperative vomiting, agitation, and hallucination were treated and recorded. The main aim of the study was to estimate the total consumption of rescue analgesics in the first six-hour postoperative period. If there was a fall in saturation postoperatively, those patients were given oxygen at a rate of 4 L/minute to maintain saturation. Those patients who had hallucinations were given injection midazolam with a minimum dose of 0.05-0.1 mg/kg to alleviate the symptoms.

## Results

**Figure 1:**
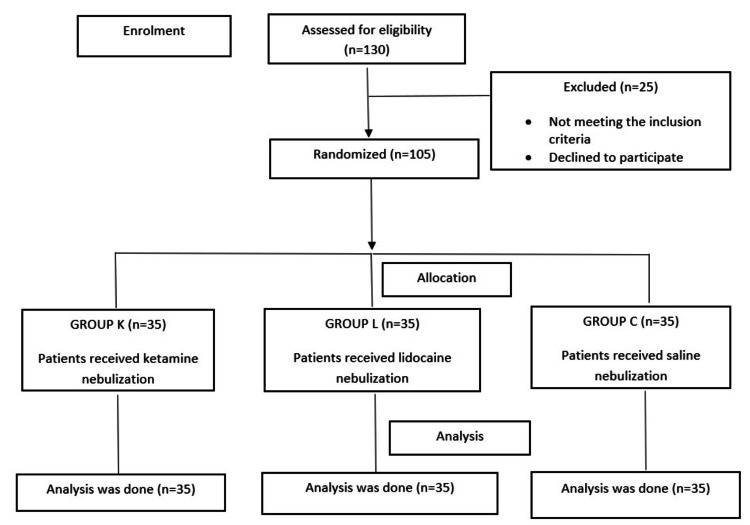
CONSORT flow diagram illustrating patient enrollment, randomization, and allocation to treatment groups n: number of patients, group K: ketamine, group L: lidocaine, group C: control, CONSORT: Consolidated Standards of Reporting Trials

In our study, we analyzed a total of 130 patients who underwent tonsillectomy during the study period. Among them, 25 patients were excluded because of reasons such as patients having upper respiratory tract infection (URTI), patients declining to participate, and age criteria not being met. Therefore, the sample size of 105 patients (divided into three groups) was selected from a total of 130 patients for postoperative pain management in tonsillectomy. The Consolidated Standards of Reporting Trials (CONSORT) guidelines have been followed in the present study (Figure 1).

Demographical data such as age, sex, and weight were found to be insignificant in all three groups since the p value is more than 0.05 (Table 1).

**Table 1:** Comparison of demographic data Group K: ketamine, group L: lidocaine, group C: control

Demographics		Group K (n=35)	Group L (n=35)	Group C (n=35)	p value
Age (years)		9.25±2.38	9.17±2.26	9.6±1.93	0.702
Gender	Male	45.7%	51.4%	40%	0.631
	Female	54.3%	48.6%	60%	
Weight		21.8±4.38	19.45±3.34	19.37±1.94	0.732

**Figure 2:**
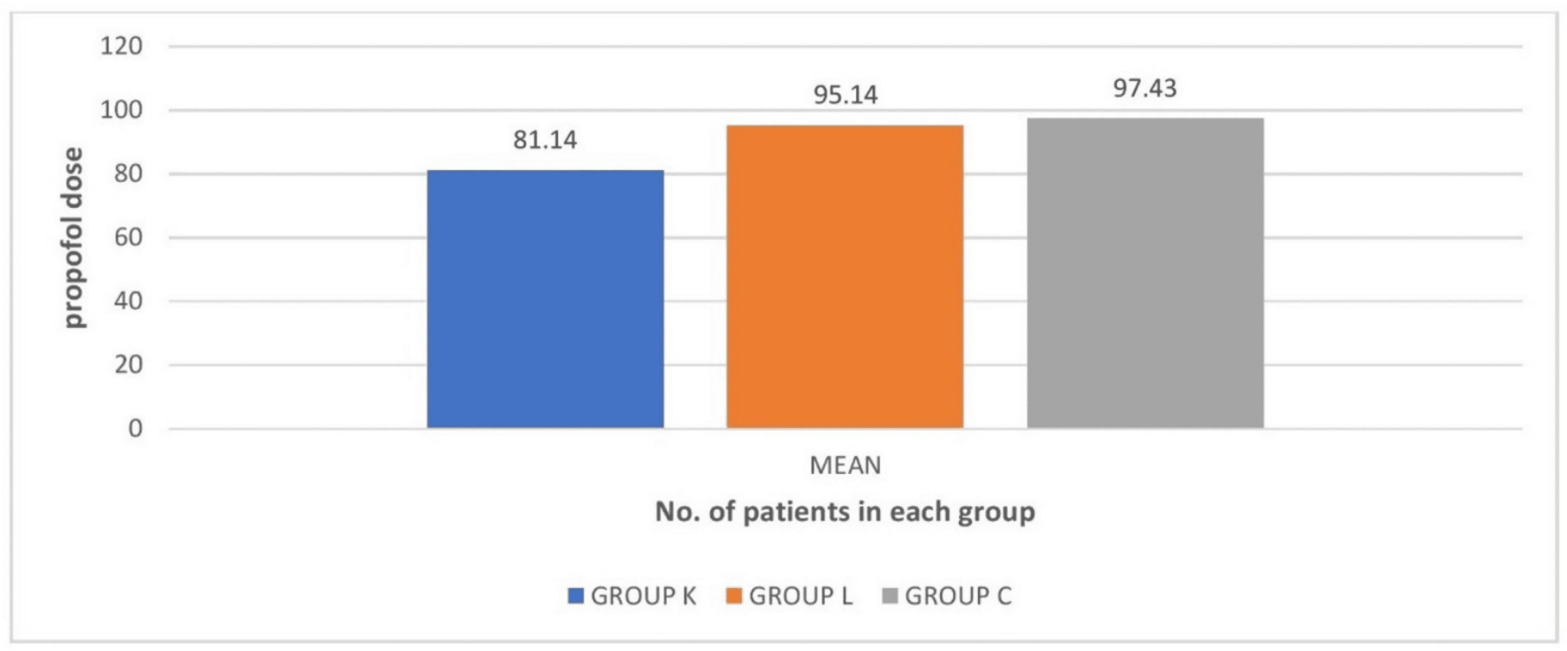
Comparison of propofol induction dose Group K: ketamine, group L: lidocaine, group C: control

When comparing the dose of propofol used during induction, it was found to be significant as the p value is less than 0.05. In groups where patients received ketamine nebulization, the dose of propofol used was lesser when compared to the other two groups (Figure 2).

**Figure 3:**
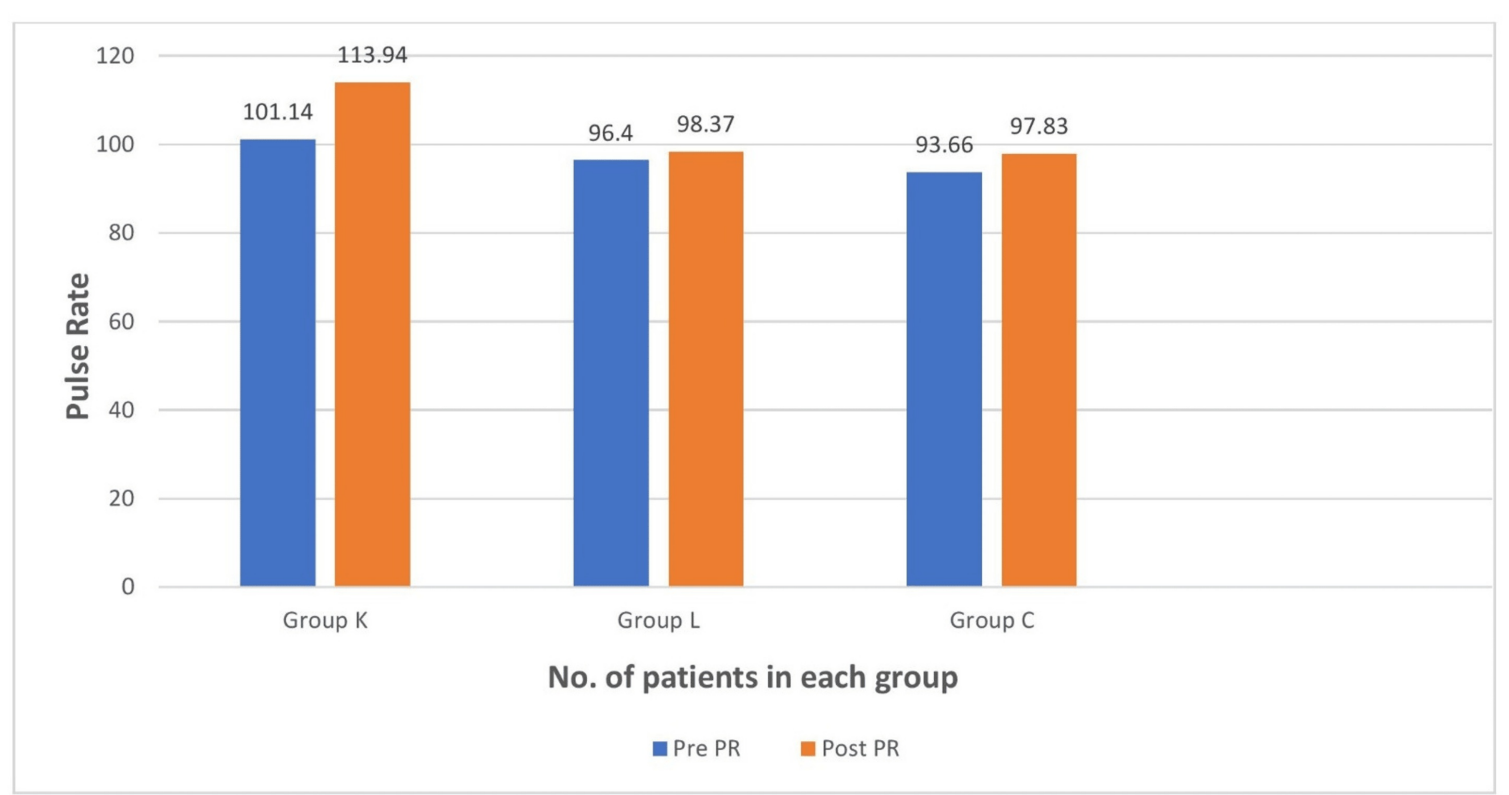
Comparison of pulse rate between the groups Group K: ketamine, group L: lidocaine, group C: control

**Figure 4:**
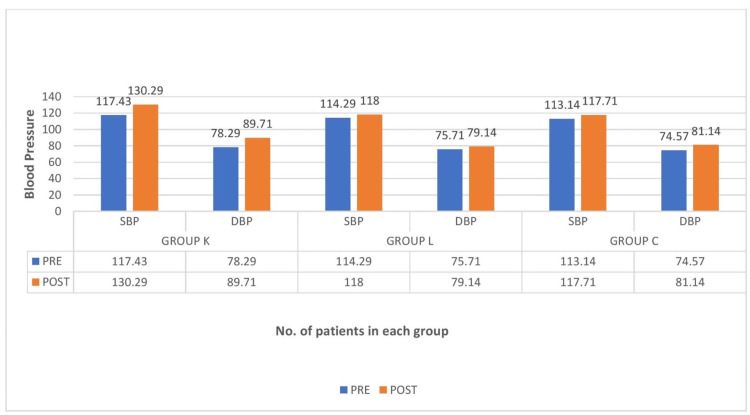
Comparison of blood pressure between the groups SBP: systolic blood pressure, DBP: diastolic blood pressure, group K: ketamine, group L: lidocaine, group C: control

The vitals of all patients were recorded before (PRE) and after (POST) nebulization. It was found to be significant with a p value of 0.001 in group K, as there was an increase in pulse rate, systolic blood pressure (SBP), and diastolic blood pressure (DBP) in most of the patients who received ketamine nebulization (Figure 3 and Figure 4).

**Figure 5:**
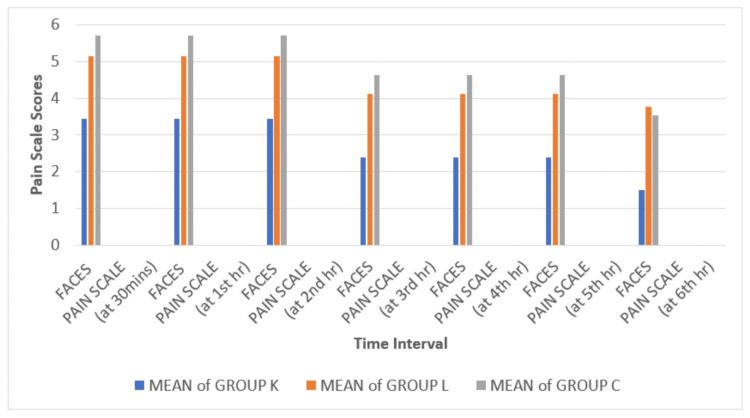
Comparison of FPS-R scores FPS-R: Faces Pain Scale-Revised, group K: ketamine, group L: lidocaine, group C: control

**Figure 6:**
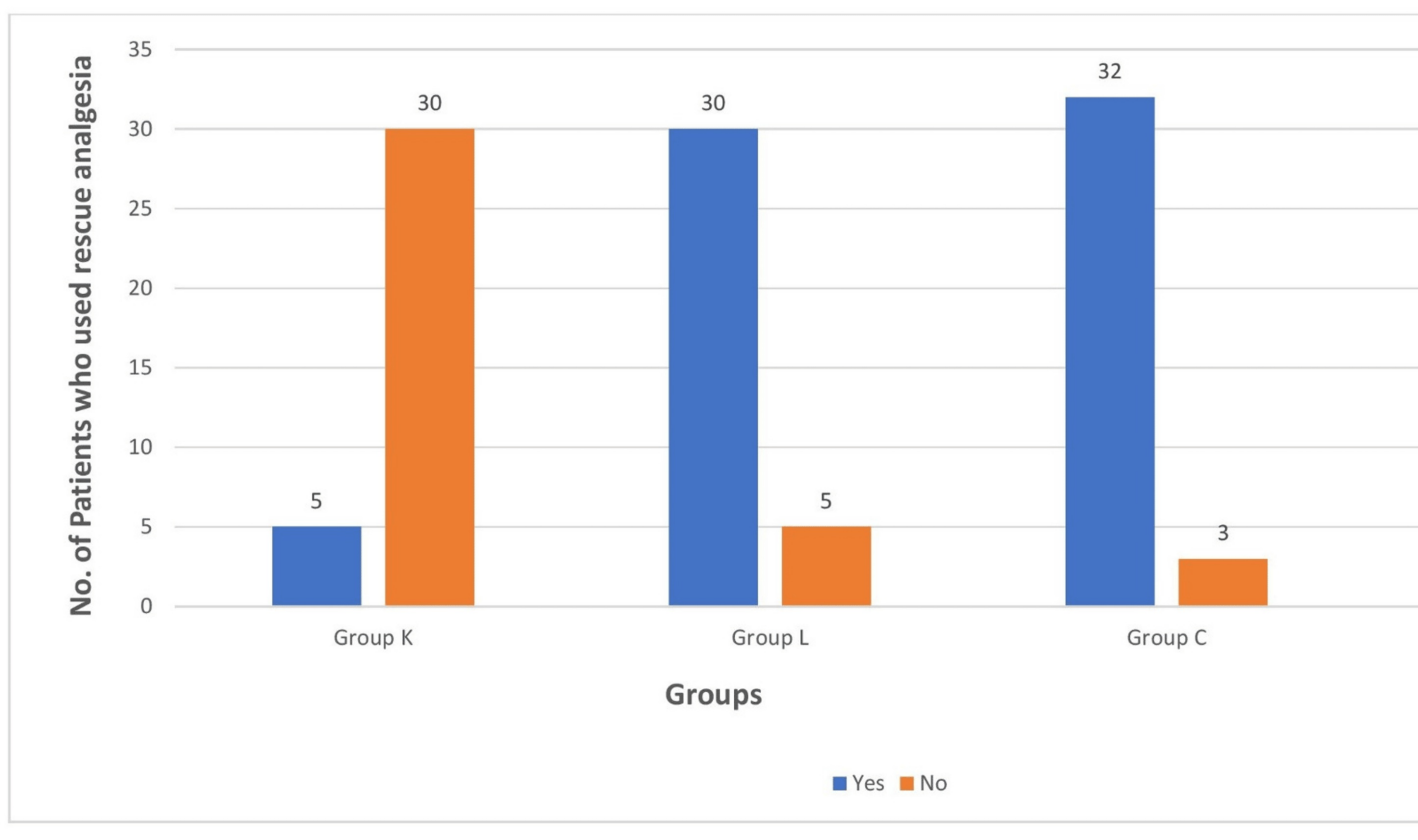
Comparison of rescue analgesia use Group K: ketamine, group L: lidocaine, group C: control

The main aim of this study is to analyze Faces Pain Scores and the usage of rescue analgesia in all three groups, which was found to be statistically significant with a p value of less than 0.05 (Table 2). In group K, where patients received ketamine nebulization, Faces Pain Scale scores were less, and the usage of rescue analgesia was comparatively lower (Figure 5 and Figure 6). Other parameters that were also noted during this study include sedation scale postoperatively and time to extubate, which were found to be statistically insignificant.

**Table 2:** Comparison of rescue analgesia usage Statistically significant as the p value is less than 0.05. Group K: ketamine, group L: lidocaine, group C: control

Gender		Number of patients	%	Chi-square test	p value
Group K	Yes	5	14.3%	56.005	<0.001
	No	30	85.7%		
Group L	Yes	30	85.7%		
	No	5	14.3%		
Group C	Yes	32	91.4%		
	No	3	8.6%		

Rescue analgesia was significantly less frequent in the ketamine group (14.3%, n=5, p<0.001) compared to the lidocaine (85.7%, n= 30) and control (91.4%, n=32) groups. Figure 5 and Figure 6 illustrate the distribution of pain scores and rescue analgesia usage across the three groups.

Postoperative complications were seen mostly in the group where patients received ketamine nebulization. A minimum number of patients complained of nausea and vomiting, and there was a fall in saturation of oxygen of less than 95% seen in group K of about 17.10% of patients, which was treated accordingly. Hallucinations were seen only in group K patients, and in the other two groups, it was not seen. These values are found to be significant since the p values are less than 0.05 (Table 3).

**Table 3:** Comparison of postoperative complications Group K: ketamine, group L: lidocaine, group C: control

Postoperative complications		Group K	Group L	Group C	p value
Nausea and vomiting	Yes	8.60%	2.90%	5.70%	0.588
	No	91.40%	97.10%	94.30%	
Desaturation	Yes	17.10%	0%	0%	0.002
	No	82.90%	100%	100%	
Hallucinations	Yes	17.10%	0%	0%	0.002
	No	82.90%	100%	100%	

## Discussion

In this study, we attempted to assess the potential benefits and duration of postoperative analgesia for tonsillectomy patients receiving pre-emptive nebulized ketamine and nebulized lidocaine. Pre-emptive administration of these medications was carried out in different studies to assess the impact on hemodynamics both during and after intubation. The main reason for using nebulization for these medications as opposed to other delivery methods was to ensure uniform and efficient drug dispersion throughout the respiratory system [11].

Our findings demonstrated that the ketamine group's induction doses of propofol were much lower, with a mean value of 81.14, than those of the lidocaine and control groups, which had mean values of 95.14 and 97.43, respectively. The findings we obtained are in accordance with the findings of Abd Ellatif et al., who discovered that intranasal ketamine considerably raised heart rate, SBP, and DBP measured during anesthesia induction at three and five minutes compared to intranasal fentanyl, with no difference at 10 and 15 minutes following intubation [12]. This might be explained by the fact that this study used a higher volume of ketamine dose than prior trials [12].

During the first six hours following surgery, the pain profile of our patients with nebulized ketamine and lidocaine exhibited considerably lower Faces Pain Scale score values than the control group. However, comparing group K and group L, there was a significant difference in Faces Pain Scale scores with group K having low pain scores, whose mean value at the sixth hour postoperative is 1.49, which is statistically significant, whereas the mean value of group L at the sixth hour is 3.77, which is slightly higher compared to group K [13].

Our results matched with those of the study by Abdel-Ghaffar et al., who reported that compared to the placebo group, the intranasal ketamine and fentanyl groups had significantly lower visual analog scale (VAS) scores in the first four hours, with an overall trend toward lower values at all time periods [14]. The decrease in Faces Pain Scale scores in our patients may be explained by the deposition of ketamine droplets in the upper airways, which result in topical analgesia and reduce inflammatory reactions in addition to the antagonistic effect of ketamine nebulization on NMDA receptors. However, systemic absorption cannot be completely ruled out.

In our study, results confirmed the postoperative analgesic effects of nebulized ketamine and lidocaine, as the time required for the request of first analgesia was significantly longer compared to the control group. The usage of rescue analgesia significantly indicates higher Faces Pain Scale scores, since the more the pain, the higher the pain scale scores and the more the usage of rescue analgesia. In comparison with lidocaine, in patients who received ketamine nebulization, the requirement of first rescue analgesia is longer. These findings are supported by the study of Abdel-Ghaffar et al., in which they observed that administering 1.5 mg/kg of pre-emptive intranasal ketamine preoperatively can significantly lessen postoperative discomfort following endoscopic nose surgery [14].

In this study, hallucinations occurred in six patients who received ketamine nebulization, and hallucinations were not seen in patients of group L and group C. This is in accordance with the study conducted by Abdel-Ghaffar et al., in which hallucinations were reported in patients who received ketamine nebulization rather than in patients who received fentanyl nebulization [14]. In this study, we found that postoperative complications such as nausea and vomiting were not statistically significant in all three groups, whereas there was a significant decrease in saturation in patients who received ketamine nebulization.

Slaton et al. noted in their study that in contrast to ketamine, which is well known to be an effective bronchodilator, nebulized lidocaine has been shown to cause early bronchoconstriction in patients with bronchial hyperactivity, such as those with chronic obstructive pulmonary disease and asthma [15]. Therefore, it is preferable to note that nebulized ketamine can function well as an alternative medication for nebulized lidocaine in the presence of such barriers [15].

The strength of this study is the use of the nebulization technique for postoperative pain management, which is a comfortable and effortless route of administration. This method of pain management is not routinely practiced, especially in tonsillectomy pediatric patients. Our findings suggest that pre-emptive nebulized ketamine offers a significant advantage in managing post-tonsillectomy pain in children, potentially reducing the need for opioid analgesics. However, the lack of ketamine plasma level data limits our understanding of its systemic effects, indicating a need for further research. More studies are required to determine the most effective time and tolerable dose for nebulization prior to intubation.

## Conclusions

Pre-emptive nebulized ketamine significantly improves postoperative pain control in pediatric tonsillectomy patients, with fewer side effects compared to lidocaine. These findings support the adoption of ketamine nebulization as a standard practice in pediatric tonsillectomy. Thus, the administration of nebulized ketamine offers an additional analgesic modality for better postoperative outcomes. Future prospective clinical trials are warranted to evaluate the safety, optimum dosing, and analgesic efficacy of nebulized ketamine in the management of postoperative painful conditions.
